# Predicted conformations of 5-HT3 receptor ion channels are modified by subunit D

**DOI:** 10.1016/j.csbj.2025.05.048

**Published:** 2025-05-29

**Authors:** Santosh T.R.B. Rao, Helen R. Irving

**Affiliations:** a La Trobe Institute for Molecular Science, La Trobe University, Bendigo, Vic 3550, Australia; b Holsworth Biomedical Research Centre, Department of Rural Clinical Sciences, La Trobe Rural Health School, La Trobe University, Bendigo, Vic 3550, Australia

**Keywords:** Serotonin, Structural Conformation, Channel Pore, Single Nucleotide Polymorphism, Transmembrane domain, Ligand binding site

## Abstract

Serotonin type 3 (5-HT_3_) receptors are ligand gated ion channels having five separate subunits (A, B, C, D, and E) that participate in formation of functional homomers (5HT3A subunit only) or heteromers with 5HT3A subunits. Since there is limited information available about the participation of the 5HT3D subunit in heteromer formation and channel pore interfaces, we explored how the 5HT3D subunit and its SNP mutated proteins contribute to conformational transitions. We generated homology models of the full-length human 5HT3D, and non-synonymous SNPs, subunits based on crystal and cryo-EM mouse 5HT3A subunit structures enabling development of heteromeric receptor models in A3D2 (A-A-D-A-D) or A3BD (A-A-B-A-D) stoichiometry. We compared these receptor models in ligand free forms or as complexes with the natural agonist serotonin or with the anti-emetic antagonist granisetron. The 5-HT bound models of the 5-HT_3_AD receptor complex display similar conformations at transmembrane and intracellular domains. However, granisetron-bound models resemble those obtained in ligand free conditions with transmembrane domains of the 5HT3D subunit showing similar conformational changes to 5HT3A subunits. Non-synonymous SNP rs6443930 (Gly 110 - Ala/Val/Asp) substitutions in the D subunit models revealed significant changes in the extracellular domain providing molecular evidence for the association of these SNPs with certain clinical disorders. Together, these data deepen our understanding of how the 5-HT3D subunit influences the gating mechanism of 5-HT_3_ receptors under ligand binding.

## Graphical Abstract

**Figure:**
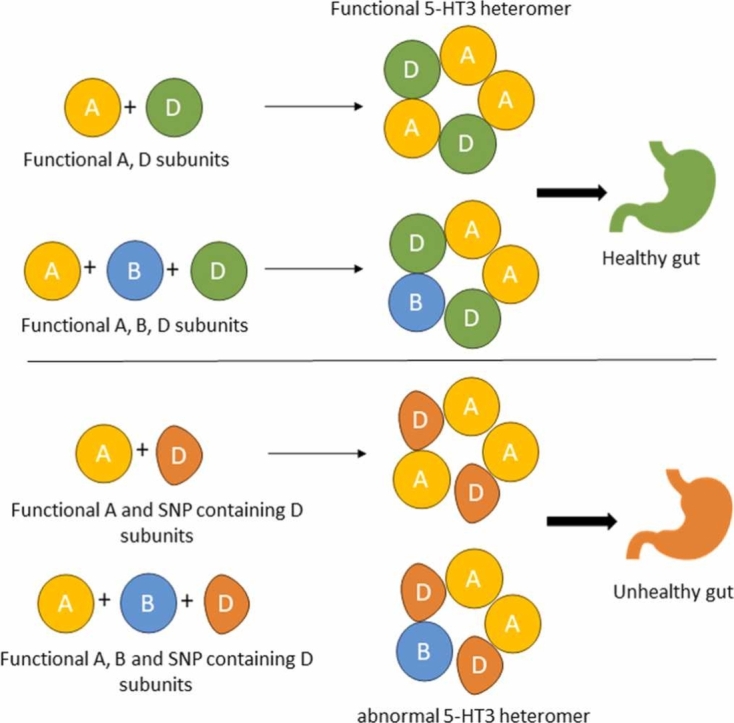

## 1 Introduction

5-hydroxytrptamine3 (5-HT_3_) receptors are pentameric ligand gated ion channels of the Cys-loop family related to nicotinic acetylcholine (nACh) and γ-aminobutyric acid (GABA) receptors [1], [2], [3]. Functionally 5-HT_3_ receptors mainly carry sodium, potassium or calcium ions following stimulation by 5-HT to mediate a rapidly activating and desensitising inward rectifying currents [1], [4], [5]. 5-HT_3_ receptors came into the limelight over 40 years ago and subunit A has been extensively explored in terms of structure and function, however, relatively little is known about the 5HT3D subunit tertiary protein structure.

Molecular simulation studies have revealed conformational changes of the 5-HT_3_ receptor pore from the closed state (impermeant to ions) to the open state (permeable to ions and water) [6], [7]. Most studies involved homomeric 5-HT_3_A receptors due to the importance of the orthosteric ligand binding site at the extracellular A+A- interface [8]. Furthermore, the 5HT3A subunit is essential to form functional receptors, either as A homomers or heteromers with other subunits. Despite the importance of the A subunit, the other subunits also participate in modulating receptor function during ligand binding. For example, various electrophysiological studies have shown that subunits B, C, D and E influence pore conductance and channel gating of heteromeric receptors [8], [9], [10], [11], [12], [13], [14], [15], [16], [17]. The complexity of human 5-HT_3_ receptors has been revealed in stages with the B subunit being identified in 1999 and then in 2003 the C, D and E subunits were recognized on chromosome 3 in humans [10], [18], [19]. Polymorphism studies have implicated all these additional subunits in various disorders and diseases [5], [20], [21]. The C, D and E subunits are less studied possibly as they do not occur in rodents [16], [22]. Of these, the D subunit is the least studied. This may be in part due to its unusual structure with a shorter extracellular domain or the fact that it is less widely expressed amongst organisms than the C or E subunits [16], [22], [23].

The human *HTR3D* gene of 7826 bp codes for a 454 amino acid long protein [12], [16], [18], [19], [23]. The human *HTR3D* gene promoter region contains binding sites for zinc finger family and RNA binding protein transcription factors [24]. Human 5HT3D subunit mRNA is widely expressed in dorsal root ganglion, colon and duodenum, kidney, and liver [16] and the protein is found in the submucosal plexus of the large intestine [12]. Single nucleotide polymorphism (SNP) studies have revealed that variants of subunit D are involved in several disorders [25]. The non-synonymous SNP rs6443930 is over represented in patients resistant to vomiting induced by anthracycline chemotherapy [26]. SNP rs6443930 has also been reported to be associated with the cleaning dimension of obsessive-compulsive disorder (OCD) in females [27] while the non-synonymous SNP rs1000952 (Arg 177- Leu/His/Pro) has been associated with OCD [28], [29]. In addition, the synonymous SNP rs12493550 was associated with primary angle closure glaucoma (PACG) [30] and SNP rs6779545 was associated with acute lymphoblastic leukemia [31]. SNPs rs6443930 and rs1000952 are non-synonymous as they are located in the coding sequence potentially effecting the protein structure, while SNPs rs1000952 and rs6779545 are situated in non-coding sequences that likely affect gene expression [28], [29], [30], [31].

The 5HT3D subunit localises at plasma membranes as a heteromer in the presence of 5HT3A subunits [16], [23]. Notably the 5HT3D subunit has a much shorter extracellular domain than the other subunits, lacking half of the Cys-loop [19], which may influence channel gating. Subtle differences are evident in responses to 5-HT and partial agonists when comparing 5-HT_3_AD receptors with 5-HT_3_AC or 5-HT_3_AE receptors [13], [16], [23]. Transmembrane (TM) regions play an important role in selective gating of the channel pore [32], [33], [34]. TM2 is particularly important as it lines the 5-HT_3_ receptor channel pore and the high sequence similarity between 5-HT_3_ receptor subunits in this region is one of their characteristic signatures [33], [34], [35]. Computational approaches investigating contributions of 5HT3D subunit in receptor function have not been reported. Therefore, we undertook to develop homology models of D subunits and incorporate these into 5-HT_3_ receptor heteromers to investigate the effects of ligand binding on pore apertures.

## 2 Modelling and methods

### 2.1 Sequence retrieval and structure modelling

All protein sequences (5HT3A, 5HT3B and 5HT3D) were retrieved from the National Centre for Biotechnology Information (NCBI) (http://www.ncbi.nlm.nih.gov; (last accessed on 16th May 2025). NP_001157118.1, a variant of subunit D was used as a sequence source for modelling. Single-particle cryo-EM structures at resolutions ranging from 3.2 Å to 4.5 Å of the mouse 5-HT_3_A homomeric receptor [36], [37], [38] were used as templates to generate human 5HT3A (AAP35868.1 - 484 amino acids), B (EAW67236.1 - 441 amino acids) and D (NP_001157118.1 - 454 amino acids) subunit models in the absence or presence of ligands [36], [37]. Complete pentamers containing just 5HT3A subunits were assembled as a reference point before assembling heteromeric 5-HT_3_ receptors. The structures were modelled and compared by using SWISS-MODEL and I-TASSER software (last accessed on 16th May 2025) and captured by UCSF ChimeraX software (last accessed 16th May 2025) [39], [40], [41].

### 2.2 Protein model comparison

In order to validate our experimentally designed 5-HT3D homology model we compared it with the human 5-HT3A (P46098) and 5-HT3D subunits (Q70Z44) protein models available in the AlphaFold data base (https://alphafold.ebi.ac.uk) [42], [43]. We choose subunit D protein isoform 3 precursor (NP_001157118.1) to derive the experimentally published structure-based model as it contains the same number of amino acids as the AlphaFold structure (Q70Z44). ChimeraX software was used to perform the comparison of protein structures and last accessed on 16th May 2025.

## 3 Results

### 3.1 Human 5HT3D subunit protein sequence variants

Four variants of human 5HT3D subunits were identified in NCBI and Ensemble databases (Supplementary Table 1). These 5HT3D subunit variants were aligned with 5HT3A subunit protein sequence (Supplementary Figure 1). Notably, all subunit D variants lack a significant number of residues in Cys-loop with three variants containing only five amino acids out of the 13 amino acid residues responsible for loop formation (Supplementary Figure 1). Two 5HT3D variants had considerably shorter extracellular domains while the other two variants (isoform 1 (NP_001138615.1) and isoform3 (NP_001157118.1)) contained similarities to the extracellular loops (A, B, C, D, E and F) present in the 5HT3A subunit. Closer inspection of these two 5HT3D variants reveals that loops B, C, D and E are lacking residues, while there is an insert in loop F, and loop A contains the same number of residues with high homology (Supplementary Figure 1). Loops D, E and F each contain one identical residue with the 5HT3A subunit while loop B has two and loop C has no identical residues (Supplementary Figure 1).

**Fig. 1:**
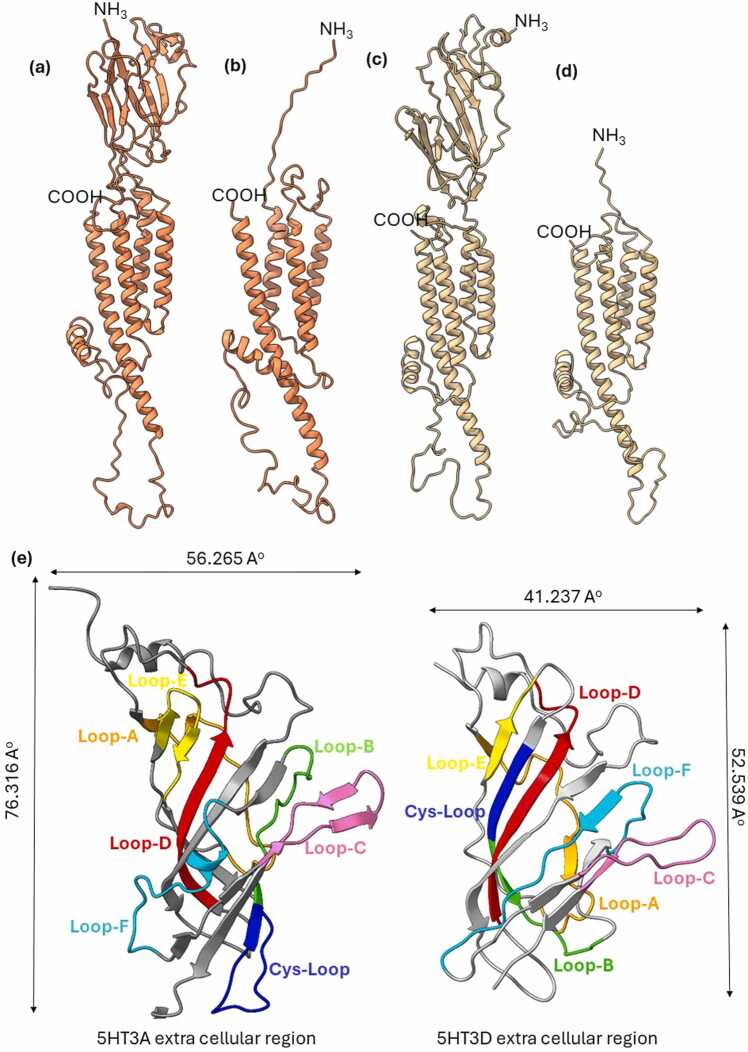
Homology models of 5-HT_3_ receptor subunit D variants. **(a)** isoform 1 NP_001138615.1; **(b)** isoform 2 NP_872343.2; **(c)** isoform 3 NP_001157118.1; **(d)** isoform x1 XP_016861343.1; **(e)** Extracellular domain from subunit A (AAP35868.1) and isoform 3 of subunit D (apo conformation) showing loop A (gold), loop B (green), loop C (pink), loop D (red), loop E (yellow), and loop F (cyan). Proteins were modelled using SWISS-MODEL [39] software and edited in UCSF Chimera software [40].

The positioning and angle of the TM domains in the variants lacking the extracellular region differs from the isoforms with extracellular domains; isoform 1 (NP_001138615.1) and isoform 3 (NP_001157118.1) (Fig. 1a-d). A conspicuous gap occurs between transmembrane domains in isoform 2 (NP_872343.2) (Fig. 1b) that potentially means this isoform cannot align at a suitable angle necessary for channel pore formation. A nearly complete lack of an extracellular domain of isoform x1 (XP_016861343.1) likely contributes to more subtle changes in its TM orientation (Fig. 1d). Isoform 1 has 7 β-strands while isoform 3 has 11 β-strands in its extracellular domain (Fig. 1a, c). These factors influenced our decision to use NP_001157118.1, a variant of subunit D (isoform 3 precursor) as a sequence source for modelling in the following receptor complexes.

Subunit D lacks half of the signature Cys-loop in its protein sequences and is shorter in the sequence size compared to other 5-HT_3_ receptor subunits [19] (Supplementary Figure 1). This lack of amino acid residues in the D subunit contributes to a smaller extracellular domain both narrower and shorter than the A subunit which is 20 Å longer (Fig. 1e). This is in part due to a condensing of the extracellular loops in the D subunit, so most of the loops merge with β-strands and there is a rearrangement of the folding pattern of loops compared to the A subunit (Fig. 1e). The Cys-loop participates in forming the interface and structural junction between extracellular domains of subunit A. Interestingly, in subunit D, this structural role is partially filled by loop B at the extracellular domain interface due to the lack of a full length Cys-loop (Fig. 1e). It is noteworthy that only one amino acid in subunit D loop B (W160) is conserved with amino acid W184 in subunit A (Supplementary Figure 1). In subunit A, this amino acid participates in forming hydrogen bonds with ligand molecules [22], [44], [45].

**Fig. 2:**
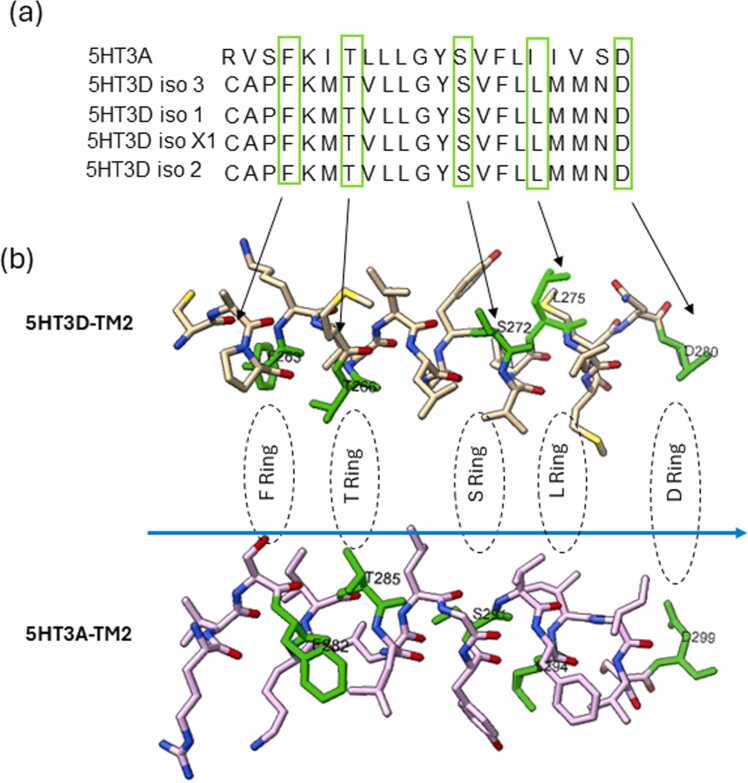
Critical residues in TM channel interface lining and pore formation in 5-HT_3_ABD heteromer. **(a)** Comparison between 5HT3A TM2 domain and subunit D variants TM2 domain protein sequence. **(b)** Composition and position of intermediate, phenylalanine, threonine, serine, leucine, aspartate, and extracellular rings along the ion channel. The sequences and coordinates are derived from homology model 5-HT_3_ABD heteromer in apo conformation. Yellow arrow denotes the direction of ion movement. The amino acid positions were assigned according to the residues studied functionally in the nicotinic acetylcholine receptor (nAChR) [48].

In contrast to the extracellular domain, all 5HT3D subunit variants have four transmembrane domains that are conserved with those in subunit A (Supplementary Figure 1). Residues in TM2 lining form the interface of channel pores playing an important role in ion movement through the channel [46]. Notably, TM2 residues in subunit D like F263, T266, S272, L275, D280 correlate to TM2 lining residues in subunit A (Fig. 2). Subunit D variants have a shorter intracellular TM3-TM4 loop than subunit A (97 versus 118 amino acids). However, the RIC-3 chaperone binding region in TM3-TM4 region responsible for 5-HT_3_ receptor translocation and cell membrane localization [47] has several identical residues in all the subunit D variants (Supplementary Figure 1).

### 3.2 Variations in protein models of subunit D variants

Homology models of the human 5HT3A, 5HT3B and 5HT3D subunits were created using the cryo-EM template of mouse 5HT3A subunit [36], [37], [38]. The homology between mouse and human 5HT3A subunits is high (87 %) and even though it falls for subunit B (44 %) and subunit D (34 %), this percentage of similarity in the protein sequence meets the requirements for modelling [49]. The homology is similar for the templates in apo, bound to the natural ligand 5-HT, and to the antagonist granisetron (Supplementary Table 2).

### 3.3 Comparison of 5HT3D models

Some differences were identified between AlphaFold models and the experimentally designed 5HT3D homology models leading to slightly different folding patterns (Supplementary Figure 2). The 5-HT3D structure modelled based on experimentally published protein model contained 429 amino acids even though we submitted 454 amino acids residues to the software. This is due to exclusion of 25 residues at the NH_3_ terminal that participate in protein colocalization at subcellular compartments and are removed during post translation modification. In the ligand binding region, β-sheets of the D subunit model developed from experimental data ranged between 2 and 6 amino acids, while β-sheets of the AlphaFold model ranged between 3 and 13 amino acids (Supplementary Figure 2b).

Transmembrane domains 1, 2, 3 and 4 show similar folding in both models based on experimental structures and AlphaFold, although TM4 in our model has a slightly inward conformation at V419. Due to this change in the bond angle at V419, the intracellular loop does not correlate with the AlphaFold model. Despite this, the β-sheets showed similar intracellular loop conformation overall (Supplementary Figure 2).

### 3.4 5-HT_3_AB, AD and ABD receptor heteromer models

Homology models of heteromeric 5-HT_3_ receptors were generated from the human models derived from the mouse 5-HT_3_A receptor homomer structures [36], [37], [38]. Since a considerable body of evidence indicates that two A subunits have to be adjacent to enable ligand binding [8], [50], we chose to create models in the A3S2 stoichiometry, where S is either two D or B subunits or a D and B subunit (Supplementary Figure 3).

Since the structures of serotonin or granisetron bound to mouse 5-HT_3_A homomeric receptors were also available (Supplementary Table 1), we generated homology models of human 5-HT_3_A homomers and superimposed the heteromers on these to determine if the D subunit impacted predicted conformations. The extracellular domains of the 5-HT_3_AB heteromer in apo and granisetron bound conformation and the 5-HT_3_AD heteromer share similar dimensions and shape. However, the width of the transmembrane domains of 5-HT_3_AB (apo), 5-HT_3_AD (apo) and 5-HT_3_ABD heteromers differ (Table 1). Interestingly, the intracellular domains of 5-HT_3_AB (apo) are greater than the diameter of 5-HT_3_A (apo) and 5-HT_3_ABD (apo) receptors (Table 1).

**Table 1:** Dimensions of the 5-HT_3_A, 5-HT_3_AB, 5-HT_3_AD, 5-HT_3_ABD receptors under different ligand bound conformations. Extracellular (EC), transmembrane (TM), and intracellular (IC) domains.

**Receptor**	**ligand bound**	**EC diameter (**Å)	**TM diameter (**Å)	**IC diameter (**Å)	**Length (**Å)
5-HT3A	Apo (ligand free)	79.641	81.797	73.120	168.301
	Granisetron	78.685	75.887	86.384	164.790
	Serotonin	78.174	82.624	84.210	165.988
5-HT_3_AB	Apo (ligand free)	80.428	76.172	73.175	168.360
	Granisetron bound	79.756	70.048	79.210	164.752
	Serotonin bound	81.400	82.551	82.187	165.933
5-HT3AD	Apo (ligand free)	79.304	81.804	73.175	165.835
	Granisetron bound	78.069	72.067	86.625	164.752
	Serotonin bound	81.817	82.762	83.489	165.752
5-HT_3_ABD	Apo (ligand free)	81.916	81.118	73.119	167.193
	Granisetron bound	78.069	76.649	86.609	164.752
	Serotonin bound	80.965	82.361	80.170	166.024

We calculated pore dimensions of the 5-HT_3_ receptors by measuring the distance between the amino acid pairs that are critical in channel pore activity. Thus, the extracellular pair is Phe 131 residue, which participates in hydrogen bond interactions with the primary ammonium group of 5-HT [22], [44], [45]. The transmembrane pair chosen is Leu 294 residue that participates in allosteric modulation of the ion channel [51]. While the intracellular pair is Arg 438 residue that is involved in single-channel conductance of the human 5-HT3A receptor [52] (Table 2). We ranked these pore dimensions from largest to smallest in the free and ligand bound states. The inclusion of the B subunit increases the pore dimensions in the extracellular pore region in the apo and granisetron bound states and transmembrane region in granisetron bound states, while the B subunit contributes to smaller pores in all states at the intracellular domain (Table 2). As subunit B has a similar transmembrane region structure to subunit A, it is possibly the lack of 13 amino acids long sequence at subunit B intracellular loop that contributes to the pore diameter differences (Supplementary Figure 4). Including the D subunit promotes larger intracellular pores in all states and contributes to a larger extracellular pore in the serotonin bound state (Table 2). Having both B and D subunits in 5-HT_3_ABD receptors generally leads to larger pores in all except the extracellular apo and intracellular serotonin bound states (Table 2). We also calculated pore dimensions of the 5-HT_3_ receptors by measuring the distance between the two amino acids of opposing subunits closest to the constricted vestibule interior (Supplementary Table 3). Similarly in this set of measurements, the inclusion of the B subunit increases the pore diameter especially in the apo and granisetron bound states. However, the effects of the D subunit are less marked and the 5-HT_3_ABD receptor pore sizes seem more aligned with those of 5-HT_3_AB receptors (Table 2 and Supplementary Table 3). Together these observations imply that the D subunit can contribute to forming proper ion channels supporting earlier electrophysiological studies [13], [16].

**Table 2:** Channel pore dimensions in different ligand bound conformations of models of 5-HT_3_A, 5-HT_3_AB_,_ 5-HT_3_AD_,_ and 5-HT_3_ABD heteromeric receptors. The distance (Å) was measured between the amino acid pairs at the interface of opposing A subunits in extracellular (EC) domain (A^+^ (Phe 131) - A^-^ (Phe 131)), transmembrane (TM) domain (A^+^ (Leu 294) - A^-^ (Leu 294)), and intracellular domain (IC) (A^+^ (Arg 438) - A^-^ (Arg 438)).

		**Pore Diameter (**Å)		
**Receptor stoichiometry**	**ligand bound**	**EC domain**	**TM domain**	**IC domain**
5-HT_3_A-A-A-A-A	Apo (ligand free)	12.754	8.079	11.044
	Granisetron bound	9.179	13.002	10.216
	Serotonin bound	10.932	17.967	11.989
5-HT_3_A-A-B-A-B	Apo (ligand free)	12.996	8.196	11.624
	Granisetron bound	10.011	14.100	10.996
	Serotonin bound	11.021	17.933	11.080
5-HT_3_A-A-D-A-D	Apo (ligand free)	12.980	8.697	11.433
	Granisetron bound	9.711	13.734	10.586
	Serotonin bound	12.002	18.064	11.646
5-HT_3_A-A-B-A-D	Apo (ligand free)	11.964	8.847	11.146
	Granisetron bound	9.989	13.762	10.631
	Serotonin bound	11.981	18.134	11.538
Summary of size differences (largest to smallest)	Apo (ligand free)	AB>AD>A>ABD	AD>ABD>AB>A	AD>ABD>A>>AB
	Granisetron bound	AB>ABD>AD>A	AB>ABD>AD>A	ABD>AD>A>>AB
	Serotonin bound	AD>ABD>AB>A	ABD>AD>A>AB	A>AD>ABD>AB

The interface between subunit A and subunit D of the 5-HT3AD receptor was investigated (Supplementary Figure 5). Fenestrations are seen in the transmembrane domains of D subunit in the apo (no ligand) and granisetron bound forms and the subunit D extracellular domain in serotonin bound conformation (Supplementary Figure 5b and 5c) with this later being due to incomplete loops and strands. The extracellular interface of 5-HT_3_AD heteromer shows a gap between the A and D subunit regions (Supplementary Figure 5c). This suggests that the conformational changes in the protein structure generates fenestrations in subunit D under different ligand bound conformations to facilitate channel pore diameter changes. However, incomplete extracellular loops of subunit D might be another reason for the fenestrations in the protein structure. TM3-TM2 loop of 5HT3D subunit changes its dimensions in both the open and closed state of the receptor (Table 1). During the open state, subunit A and D TM3-TM4 loop condensed outwards facilitating the opening of the intracellular domain, while the apo or granisetron bound models have similar conformation to the closed state (Supplementary Figure 6 and Supplementary Figure 7).

### 3.5 Effects of ligands on the 5-HT_3_ABD heteromer

Developing the 5-HT_3_ABD heteromer (apo conformation) model involved superimposing three A, one B, and one D monomers on the 5-HT_3_A homomeric structure using UCSF-ChimeraX software. Interestingly, a large gap between subunit D and subunit A extracellular domain interface was observed (Supplementary Figure 5), due to the smaller diameter of the subunit D extracellular domain (Fig. 1e). Notably, subunit B shows a clear alignment with subunit A at the extracellular domain interface. However, the extracellular domain of the subunit D appears incomplete in the 5-HT_3_ABD heteromer space filling model compared to subunit A and B (Supplementary Figure 3). Despite this, a channel pore interface appears aligned with both subunit B and D. The TM2 domain lining interface residues in subunit D are identical to subunit A and the channel lining show these residues arranged in face-to-face manner in the heteromer (Fig. 2). Possibly this conserved transmembrane domain of subunit D and subunit A helps in the channel pore formation and notionally supports ion movement through lipid bilayers (Fig. 2).

**Fig. 3:**
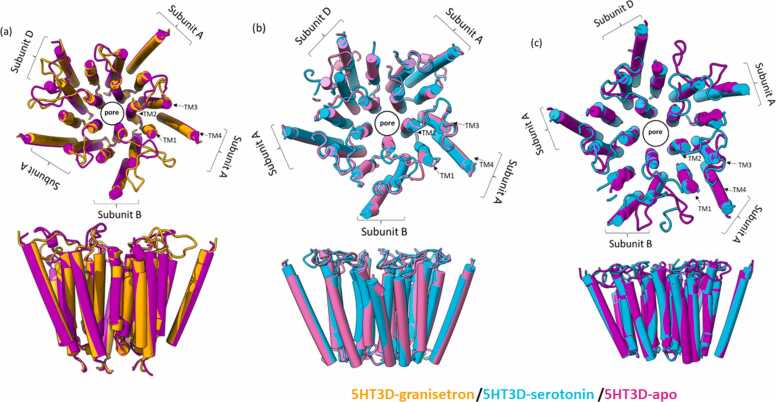
Pairwise overlays of the TM domain illustrating transitions at the quaternary level of 5-HT_3_ receptors. Models were superimposed on the 5-HT_3_A receptor homomer. Overlay images represent the movement of the TM domains when granisetron or serotonin are bound. 5-HT_3_ABD heteropentamer overlay of **(a)** granisetron bound (orange) and apo conformation (magenta), **(b)** serotonin bound (cyan) and apo conformation (hot pink) and **(c)** serotonin bound (cyan) and granisetron bound (magenta).

Mouse 5-HT_3_A receptor homomer in apo conformation studies is 165 Å long with a diameter of 80 Å [36], and models of the human 5-HT_3_A receptor homomer apo conformation have similar dimensions of 79.64 Å extracellular diameter and 168.30 Å length. The human 5-HT_3_ABD heteromer in apo conformation is 167.19 Å long with a diameter of 81.92 Å (Table 1). Models with bound ligands such as granisetron or serotonin show different conformations that impact on pore dimensions (Table 2 and Supplementary Figure 8). When serotonin is bound, this causes the TM domains to move towards each other slightly, thus the channel pore of the intracellular region opens and allows the ions through (Table 2 and Fig. 3). However, for the ligand free apo and granisetron-bound conformations, the distance between TM2 domains and intracellular domain are relatively similar, thereby restricting ion flow. Interestingly, binding of serotonin is predicted to result in larger pore dimensions in ABD heteromers than with either AB or A receptors except at the intracellular domain where the A homomer has the largest pore (Table 2).

### 3.6 Non-synonymous SNPs in subunit D modify the protein structure

**Fig. 4:**
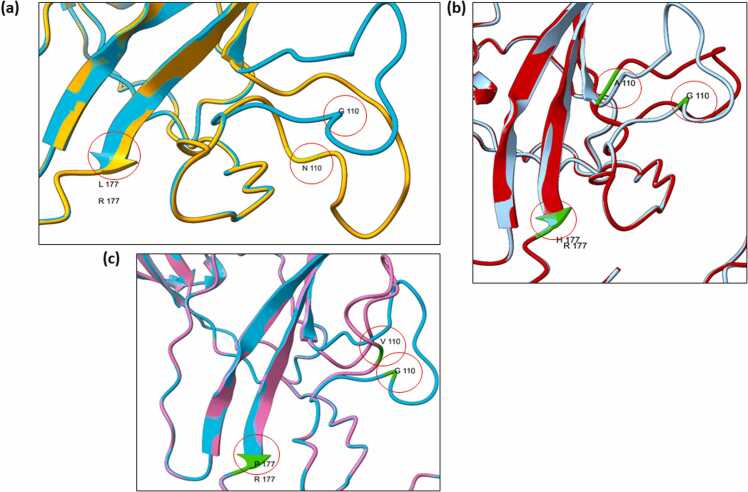
Non-synonymous SNPs rs1000952 (at Gly110) and rs6443930 (at Arg177) influence subunit D protein models in the extracellular region. **(a)** SNP substitution at Gly110-Asp110 and Arg177-Leu177; **(b)** SNP substitution at Gly110-Ala110 and Arg177-His177; **(c)** SNP substitution at Gly110-Val110 and Arg177-Pro177. The 5HT3D (NP_001138615.1) protein model without SNPs was developed using AlphaFold structure Q70Z44 as a template and represented in cyan. SNP substitution Asp110, Leu177 model is in orange; SNP substitution Ala110, His177 model is in red; SNP substitution Val110, Pro177 model is represented in hot pink; and SNP amino acid positions highlihgted by red circles on the models.

Non-synonymous SNPs rs6443930 (Gly 110) and rs1000952 (Arg 177) occur in the coding region of the extracellular domain of 5HT3D subunit (NP_001138615.1). Several possible amino acid substitutions can occur, and the position of these SNPs differs depending on the D subunit variants used (e.g. Gly 36 in subunit D variant NP_872343.2 is the same as Gly 110 in subunit D variant NP_001138615.1; Supplementary Table 4). Homology models of these possible non-synonymous SNP substitutions were generated with 5-HT3D (NP_001138615.1) protein sequence (Fig. 4). Based on these models, SNP rs1000952 (Arg 177-His/Pro/Leu) substitutions show few differences in the folding of the extracellular domain (Fig. 4). However, SNP rs6443930 (Gly 110- Ala/Val/Asp) amino acid substitutions show considerable deviations in alignment of the loops in the extracellular domain of the protein model (Fig. 4). To further investigate this, we created ABD heteromer models with SNP rs1000952 (Arg 177-His/Pro/Leu) substitutions (Supplementary Figure 9). These models all show a condensed extracellular domain of subunit D leading to gaps between the associated A subunits (Supplementary Figure 9). Furthermore, significant changes in the subunit D structural axis can be observed at extracellular, transmembrane and intracellular domains (Supplementary Figure 9).

## 4 Discussion and conclusions

Subunit D is an understudied member of the 5-HT_3_ receptor family due to it lacking parts of the extracellular domain and intracellular loop leading investigators to discount it. Additionally, subunit D has a relatively limited expression across different animal species [16], [22]. Another reason contributing to fewer studies is that subunit D lacks the signal peptide essential for localising to plasma membranes. It has been suggested that the D subunit reaches the plasma membrane in association with subunits like the A subunit that contain appropriate signal peptides [19]. Despite these factors, several studies have implicated that polymorphisms in subunit D contribute to various disorders in humans [26], [28], [30], [31] thereby underscoring the importance of all the different 5-HT_3_ receptor subunits. Electrophysiological studies using transfected cells have also indicated that the D subunit subtly alters ion channel characteristics of 5-HT_3_ receptors [13], [16], while it is well established that the B subunit markedly influences 5-HT_3_ receptor function [9], [16], [53], [54], [55]. Subunit D is 35.5 % homologous in its protein sequence with subunit A [19]; and this is much higher in transmembrane regions which are highly conserved in all 5-HT_3_ receptor subunits including all four D variants. Here we employed the recent high-resolution structures of 5-HT_3_A receptor to create homology models incorporating the D subunit and compared these with models including the B subunit. These models reveal that the D subunit is likely to modify pore sizes in heteromeric receptors in a different fashion to the B subunit.

It is well established that several residues in subunit A extracellular domain are important for ligand recognition and ligand binding through hydrogen bonding and van der Waals forces [15], [36]. Subunit B, C, and E proteins have full length extracellular domains with many of the critical residues present in the A subunit. Thus, extracellular regions of these B, C and E subunits are important in forming a structurally complete heteromer [8], [22]. While subunit D lacks several components of the extracellular domain and half the Cys-loop and is generally smaller in extracellular region specially at areas aligned to the orthosteric binding site [16], [19]. In addition, subunit D lacks the residues found in subunit A (e.g. Y74, F131, S164, and D166) participating in orthosteric or allosteric site formation [56], [57]. These observations preclude the possibility of a ligand binding interface forming between subunit A and subunit D.

The TM2 domain lines the channel pore and residues in the subunit D TM2 are conserved with the subunit A residues. Channel opening is regulated by binding at orthosteric sites and modulated by allosteric binding and together these in turn drive conformational changes in the TM2 lining. Specific residues from the TM2 lining regulate movement of ions in the pore; these residues interact with the same residues in the opposing subunit to create a uniform pore diameter [32], [46]. Models incorporating subunit D are predicted to participate in formation of the channel pore interface similarly to 5-HT_3_A homomeric receptors. Like other members of the ligand gated ion channels such as nACh receptors [48], residues in 5-HT_3_ABD transmembrane domain interface form a series of five rings (F, T, S, L, and D). These observations imply that our models of heteromers containing subunit D can form functional channel pores with TM2 channel lining facilitating ion passage. Such an interpretation is supported by prior electrophysiological studies demonstrating subtle effects of the D subunit on 5-HT_3_AD heteromers compared to 5-HT_3_A homomer receptor function [13], [16], [23]. However, fenestrations occur in the extracellular domain regions of subunit D in the apo and granisetron bound models. Fenestration were also observed in TM domain region of subunit D in serotonin bound conformations. This could be due to the smaller size of the extracellular domain and intracellular loop of subunit D compared to other subunits. Therefore, it was pertinent to see if models containing subunit D could form similar sized pores.

Stoichiometry of the heteromeric receptor plays crucial roles in forming channel pores and dictates the functionality of the receptor. Pharmacological and biophysical studies have revealed that 5-HT_3_ receptors have a A3S2 stoichiometry for 5-HT_3_AB receptors [50], [58]. This stoichiometry is presumed to also apply to the other diheteromers and likely to triheteromers as A+ , A- interface is essential to generate a ligand binding domain [8], [35].

5-HT_3_AD heteromeric receptor (A-A-D-A-D) pore dimensions differ to those of the 5-HT_3_AB heteromer, with the 5-HT_3_AD being larger at the intracellular domain. The 5-HT_3_ABD heteromers have a larger pore size at transmembrane domain compared to 5-HT_3_AD and 5-HT_3_AB heteromers in 5-HT bound conformation. Overall, the pore dimensions of the 5-HT_3_ABD model are generally larger than the 5-HT_3_A homomeric receptor in the apo and granisetron bound state. These predictions thus provide some modelling evidence to support the subtle changes reported in electrophysiological studies involving the D subunits where it has some effect on diheteromeric 5-HT_3_ receptors that are nowhere as marked as the B subunit [13], [16], [47], [48]. This observation is interesting as the only report examining a tri-heteromeric 5-HT_3_ receptor (the ACE heteromer) at the functional level reveals a dramatic shift in the power of palonosetron to antagonise the receptor compared to the di-heteromers AC or AE [14].

While SNPs in subunit D are associated with disorders in humans, yet there is no clear evidence how these SNPs participate in these conditions. The non-synonymous SNPs rs6443930 (Gly 110 - Ala/Val/Asp) substitutions in the D subunit showed significant changes in the extracellular domain of the homology models. A 17 amino acid sequence from the Ser106 through to the Thr123 is affected and deviates from its original folding due to the SNP substitution at Gly 110. This SNP is located within the extracellular region that forms a ligand binding interface of the heteromeric receptor. This is an important consideration as this SNP is associated with reduced chemotherapy induced nausea and vomiting (CINV) following treatment with ondansetron in patients with breast cancer [26]. Further, no associations with SNPs in A or B subunits have been reported for CINV in cancer patients treated with 5-HT_3_ receptor antagonists as antiemetics [26], [59], [60], [61] underlining the importance of these substitutions in the D subunit. SNP rs6443930 has also been associated with cleaning dimensions (contamination/cleaning) of OCD in females [27] possibly underscoring that D heteromers contribute to gender based clinical responses. As it is relevant to the formation of heteromers, the non-synonymous SNP rs1176744 (Tyr129Ser) of the B subunit that increases opening times of AB receptors is associated with many conditions including depression and OCD [5], [20], [26], [27].

The other non-synonymous SNP of the D subunit rs1000952 (Arg 177- Leu/His/Pro) has also been weakly associated with OCD [28], [29]. Perhaps surprisingly no significant folding changes were detected in our models although arginine with a positive side chain is replaced with neutral amino acid, leucine or proline. SNP rs1000952 occurs at the end of a beta fold where the changes to leucine or proline may not be so marked while subtle changes are seen with the histidine residue, and these could be more marked pending its ionization state during pH changes in situ. This part of the extracellular domain may be more susceptible to various changes in pH in the extracellular matrix with histidine residue side chain having a pK_a_ of 6.04. 5HT3D subunits have been detected in gut epithelial layers [12] where food movement accompanies changes in lumen pH that may influence conformation of receptors containing D subunits with histidine and or aspartic acid SNPs that possibly lead to abnormalities in the receptor function.

## 5 Limitations

The major limitation of this study is that it is entirely based on homology models of heteromeric receptors with subunit D. Although we compared our models of subunit A with AlphaFold predictions and observed similar α-helices and β-sheets throughout the receptor structure, the predictions of models based on weaker homology are less reliable. Despite this limitation, our models did reveal that the D subunit is likely to modify pore sizes in heteromeric receptors in a different fashion to AB heteromers. Nevertheless, these predictions from modelling studies require validation in molecular dynamic simulations in the first instance. These should be accompanied by follow up cell-based studies where the expression of D and other subunits can be controlled. The present study provides a basis on which to plan cell-based experiments to study how the D subunit influences the 5-HT_3_ receptor activity.

## CRediT authorship contribution statement

**Santosh T. R. B. Rao:** Writing – review & editing, Writing – original draft, Visualization, Validation, Methodology, Investigation, Formal analysis, Data curation, Conceptualization. **Helen R. Irving:** Writing – review & editing, Visualization, Supervision, Project administration, Methodology, Funding acquisition, Conceptualization.

## Declaration of Competing Interest

None.
