# Supplementary material for: Predicted conformations of 5-HT3 receptor ion channels are modified by subunit D
Source: Comput Struct Biotechnol J. 2025 May 29;27:2394–402. doi: 10.1016/j.csbj.2025.05.048 (PMC12172986; doi:10.1016/j.csbj.2025.05.048)
Supplement: Supplementary file 1 — Supplementary material [file mmc1.docx]

**Supplementary Figures for**

**Predicted conformations of 5-HT3 receptor ion channels are modified by subunit D**

**Santosh T. R. B. Rao^1,2^, Helen R. Irving^1,2^**

^1^ La Trobe Institute for Molecular Science, La Trobe University, Bendigo Vic 3550, Australia.

^2^ Holsworth Biomedical Research Centre, Department of Rural Clinical Sciences, La Trobe Rural Health School, La Trobe University, Bendigo Vic 3550, Australia.

**List of Figures**

**Supplementary Figure 1** Clustal Omega alignment of human 5-HT_3_ receptor subunit A with the D subunits:

**Supplementary Figure 2**. Comparison of subunit D models from AlphaFold (pink) and experimentally modelled structure in SWISS-MODEL (cyan).

**Supplementary Figure 3** Subunit interface and channel pore variations in homology models of 5-HT_3_A homopentamer and 5-HT_3_ABD heteropentamers in apo conformation.

**Supplementary Figure 4** Clustal Omega alignment of human 5-HT_3_ receptor subunits 5HT3A (AAP35868.1), 5HT3B (EAW67236.1), 5HT3D isoform 1 precursor (NP_001138615.1).

**Supplementary Figure 5** Interface of heteromeric 5-HT_3_AD receptors.

**Supplementary Figure 6.** Comparison of conformational changes in subunit A of apo, granisetron and serotonin bound conformations.

**Supplementary Figure 7.** Comparison of conformational changes in homology models of subunit D.

**Supplementary Figure 8** Comparison of heteromeric 5-HT3 receptors.

**Supplementary Figure 9** Comparison of ABD heteromer with subunit D SNP substituted models.

AAP35868.1 MLGKLAMLLWVQQALLALLLPTLLAQGEARRSRN-T--TRPALLRLSDYLLTNYRKGVRP 57

NP_001157118.1 -MQK----HSPGPPALALLSQSLLTTGNGDTLIINCPGFGQHRVDPAAFQAVFDRKAIGP 55

NP_001138615.1 ----------------------------------------------------MER----- 3

NP_872343.2 ------------------------------------------------------------ 0

XP_016861343.1 ------------------------------------------------------------ 0

AAP35868.1 VRDWRKPTTVSIDVIVYAILNVDEKNQVLTTYIWYRQYWTDEFLQWNPEDFDNITKLSIP 117

NP_001157118.1 VTNYSVATHVNISFTLSAIWNCYSRIHT--FNCHHARPWHNQFVQWNPDECGGIKKSGMA 113

NP_001138615.1 --GWFH----GKGFLLGFILHLLLQDSH--LQLVTSFLW---LNMWNPDECGGIKKSGMA 52

NP_872343.2 ------------------------------------------------------------ 0

XP_016861343.1 ------------------------------------------------------------ 0

AAP35868.1 TDSIWVPDILINEFVDVGKSPNIPYVYIRHQGEVQNYKPLQVVTACSLDIYNFPFDVQNC 177

NP_001157118.1 TENLWLSDVFIEESVDQTPAGLMASMSIVKAT-------SNTISQCGWSA---------- 156

NP_001138615.1 TENLWLSDVFIEESVDQTPAGLMASMSIVKAT-------SNTISQCGWSA---------- 95

NP_872343.2 ----------------------MASMSIVKAT-------SNTISQCGWSA---------- 21

XP_016861343.1 ------------------------------------------------------------ 0

AAP35868.1 SLTFTSWLHTIQD---------------INISLWRLPEK--VKSDRSVFMNQGEWELLGV 220

NP_001157118.1 ---SANWTPSISPSMDRA-------------RAWRRMSRSFQIHHRTSFRTRREWVLLGI 200

NP_001138615.1 ---SANWTPSISPSMDRGERSPSALSPTQVTRAWRRMSRSFQIHHRTSFRTRREWVLLGI 152

NP_872343.2 ---SANWTPSISPSMDRGERSPSALSPTQ------------------------------- 47

XP_016861343.1 ----------------------------M------------------------------- 1

AAP35868.1 LPYFREFSMESSNYYAEMKFYVVIR--RRPLFYVVSLLLPSIFLMVMDIVGFYLPPNSGE 278

NP_001157118.1 QKRTIKVTVATN-QYEQAIFHVAIRRRCRPSPYVVNFLVPSGILIAIDALSFYLPLESGN 259

NP_001138615.1 QKRTIKVTVATN-QYEQAIFHVAIRRRCRPSPYVVNFLVPSGILIAIDALSFYLPLESGN 211

NP_872343.2 ---------------------VAIRRRCRPSPYVVNFLVPSGILIAIDALSFYLPLESGN 86

XP_016861343.1 ---------------------VAIRRRCRPSPYVVNFLVPSGILIAIDALSFYLPLESGN 40

*.** ** ***.:*:** :*:.:* :.**** :**:

AAP35868.1 RVS**F**KI**T**LLLGY**S**VFL**I**IVS**D**TLPATAIG--------------TPLIGVYFVVCMALLVI 324

NP_001157118.1 CAP**F**KM**T**VLLGY**S**VFL**L**MMN**D**LLPATSTSSHASLVAPLALMQTPLPAGVYFALCLSLMVG 319

NP_001138615.1 CAP**F**KM**T**VLLGY**S**VFL**L**MMN**D**LLPATSTSSHASLVRPHPSR--DQKRGVYFALCLSLMVG 269

NP_872343.2 CAP**F**KM**T**VLLGY**S**VFL**L**MMN**D**LLPATSTSSHASLVRPHPSR--DQKRGVYFALCLSLMVG 144

XP_016861343.1 CAP**F**KM**T**VLLGY**S**VFL**L**MMN**D**LLPATSTSSHASLVRPHPSR--DQKRGVYFALCLSLMVG 98

. **:*:**********:**::.***** ****: . ****.:*::*:*

AAP35868.1 SLAETIFIVRLVHKQDL-QQPVPAWLRHLVLERIAWLLCLREQSTSQRPPATSQATKTDD 383

NP_001157118.1 SLLETIFITHLLHVATTQPLPLPRWLHSLLLHCTGQGRCCPTAPQKG--------N---- 367

NP_001138615.1 SLLETIFITHLLHVATTQPLPLPRWLHSLLLHCTGQGRCCPTAPQKG--------N---- 317

NP_872343.2 SLLETIFITHLLHVATTQPLPLPRWLHSLLLHCTGQGRCCPTAPQKG--------N---- 192

XP_016861343.1 SLLETIFITHLLHVATTQPLPLPRWLHSLLLHCTGQGRCCPTAPQKG--------N---- 146

** *****.:*:* *:* **: *:*. . * . .

AAP35868.1 CSAMGNHCSHMGGPQDFEKSPRDRCSPPPPPREASLAVCGLLQELSSIRQFLEKRDEIRE 443

NP_001157118.1 -KGPGLTPTHLPGVKE----PEVSAGQMPGPGEAELTGGS-----EWTRAQREHEAQKQH 417

NP_001138615.1 -KGPGLTPTHLPGVKE----PEVSAGQMPGPGEAELTGGS-----EWTRAQREHEAQKQH 367

NP_872343.2 -KGPGLTPTHLPGVKE----PEVSAGQMPGPGEAELTGGS-----EWTRAQREHEAQKQH 242

XP_016861343.1 -KGPGLTPTHLPGVKE----PEVSAGQMPGPGEAELTGGS-----EWTRAQREHEAQKQH 196

.. * :*: * :: *. .. * * **.*: . . * *:. : :.

AAP35868.1 VARDWLRVGSVLDKLLFHIYLLAVLAYSITLVMLWSIWQYA 484

NP_001157118.1 SVELWVQFSHAMDALLFRLYLLFMASSIITVICLWNT---- 454

NP_001138615.1 SVELWVQFSHAMDALLFRLYLLFMASSIITVICLWNT---- 404

NP_872343.2 SVELWVQFSHAMDALLFRLYLLFMASSIITVICLWNT---- 279

XP_016861343.1 SVELWVQFSHAMDALLFRLYLLFMASSIITVICLWNT---- 233

.. *::.. .:* ***::*** : : **:: **.

**Supplementary Figure 1** Clustal Omega alignment of human 5-HT_3_ receptor subunit A with the D subunits: 5HT3A (AAP35868.1), 5HT3D isoform X1 (XP_016861343.1), 5HT3D isoform 1 precursor (NP_001138615.1), 5HT3D isoform 3 precursor (NP_001157118.1) and 5HT3D isoform 2 (NP_872343.2). The highlights represent: Cys-loop (yellow), TM1 (green), TM2 (cyan), TM3 (pink), and TM4 (red) domains; with RIC-3 binding region in the intracellular TM3-TM4 loop (teal) and loops in extra cellular region (grey). The order of the extracellular loops in grey from amino terminal to carboxy terminal is as follows D, A, E, B, F, and C. Conserved F, T, S, L and D residues in TM2 lining are in red and SNP residue substitution positions are in yellow and highlighted in army green. The asterisk (*), colon (:), and dot (.) indicate identical amino acid residues, conserved substitutions, and semi-conserved substitutions across all sequences used in the alignment, respectively.


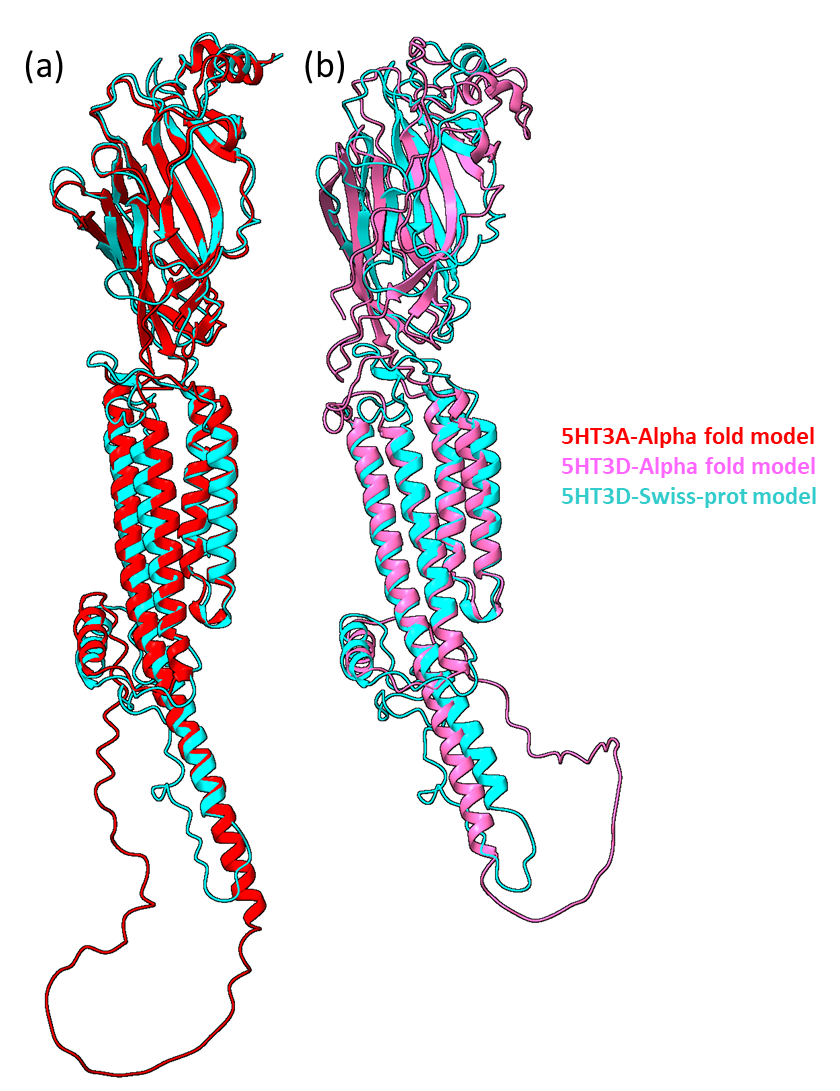


**Supplementary Figure 2** Comparison of AlphaFold models and models based on experimental structures. **(a)** Subunit A models from AlphaFold (red) and experimentally modelled subunit D (NP_001157118.1) in SWISS-MODEL (cyan). **(b)** Subunit D models from AlphaFold (pink) and experimentally modelled in SWISS-MODEL (cyan).


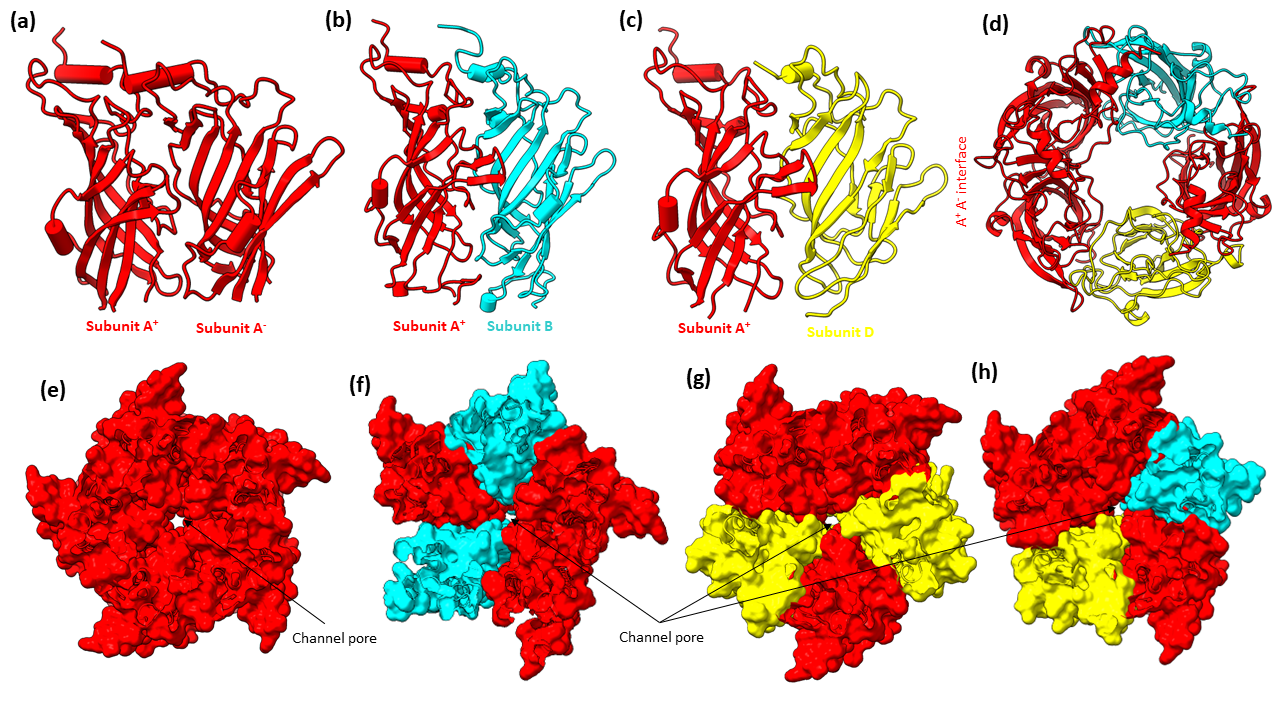


**Supplementary Figure 3** Subunit interface and channel pore variations in homology models of 5-HT_3_A homopentamer, 5-HT_3_AB, 5-HT_3_AD and 5-HT_3_ABD heteropentamers in apo conformation. Protein models of subunit A coloured red, subunit B in cyan and subunit D in yellow. Models of the extracellular domain of subunit A^+^ A^-^ interface **(a)**; subunit A^+^ B interface **(b)**; and subunit A^+^ D interface **(c)**. Ribbon model of the A-A-B-A-D heteromer **(d)**. Space filling models of channel pores of 5-HT_3_A receptor homopentamer **(e)** 5-HT_3_AB heteropentamer **(f)**; 5-HT_3_AD heteropentamer **(g)**; and 5-HT_3_ABD heteropentamer **(h)**.

NP_001138615.1 ---------------------------------------------------------MER 3

AAP35868.1 MLGKLAMLLWVQQALLALLLPTLLAQGE-ARRSRNTTRPALLRLSDYLLTNYRKGVRPVR 59

EAW67236.1 MLSSVMAPLWACIL---------VAAGILATDTHHPQDSALYHLSKQLLQKYHKEVRPVY 51

NP_001138615.1 GWFHGKGFLLGFILHL-LLQDSHLQLVTSFLWLN--------MWNPDECGGIKKSGMATE 54

AAP35868.1 DWRKPTTVSIDVIVYAILNVDEKNQVLTTYIWYRQYWTDEFLQWNPEDFDNITKLSIPTD 119

EAW67236.1 NWTKATTVYLDLFVHAILDVDAENQILKTSVWYQEVWNDEFLSWNSSMFDEIREISLPLS 111

.* : . . :..::: * * . *::.: :* . ** . . * : .: .

NP_001138615.1 NLWLSDVFIEESVDQTPAGLMASMSIVKAT-------SNTISQCGWSASANWTPSISPSM 107

AAP35868.1 SIWVPDILINEFVDVGKSPNIPYVYIRHQGEVQNYKPLQVVTACSLDIYN-F------PF 172

EAW67236.1 AIWAPDIIINEFVDIERSPDLPYVYVNSSGTIENYKPIQVVSACSLETYA-F------PF 164

:* *::*:* ** : : : : :.:: *. . : :

NP_001138615.1 DRGERS---PSALSP-TQVTRAWRRMSRSFQIHHRTSFRTRREWVLLGIQKRTIKVT-VA 162

AAP35868.1 DVQNCSLTFTSWLHTIQDINISLWRLP-EKVKSDRSVFMNQGEWELLGVLPYFREFSMES 231

EAW67236.1 DVQNCSLTFKSILHTVEDVDLAFLRSP-EDIQHDKKAFLNDSEWELLSVSSTYSILQ-SS 222

* : * * * :: : * . .:. * . ** **.: . :

NP_001138615.1 TNQYEQAIFHVAIRRRCRPSPYVVNFLVPSGILIAIDALSFYLPLESGNCAP**F**KM**T**VLLG 222

AAP35868.1 SNYYAEMKFYVVIRR--RPLFYVVSLLLPSIFLMVMDIVGFYLPPNSGERVS**F**KI**T**LLLG 289

EAW67236.1 AGGFAQIQFNVVMRR--HPLVYVVSLLIPSIFLMLVDLGSFYLPPNCRARIV**F**KT**S**VLVG 280

:. : : * *.:** :* ***.:*:** :*: :* .**** :. ****** **:**:*:*

NP_001138615.1 YSVFLLMMNDLLPATSTSSHASLVRPHPSRDQKRGVYFALCLSLMVGSLLETIFITHLLH 282

AAP35868.1 YSVFLIIVSDTLPATAIGT------------PLIGVYFVVCMALLVISLAETIFIVRLVH 337

EAW67236.1 YTVFRVNMSNQVPRSVGST------------PLIGHFFTICMAFLVLSLAKSIVLVKFLH 328

*:** : :.: :* : .: * :*.:*::::* ** ::*.:.:::*

NP_001138615.1 VATTQPLPLPRWLHSLLLHCTGQGRCCPTAPQKGNKGPGLTPT--------------HL- 327

AAP35868.1 KQDLQ-QPVPAWLRHLVLERIAWLLCLREQST--SQRPPATSQATK--TDDCSAMGNHCS 392

EAW67236.1 DEQRG-GQE------------QPFLCLRGDTD--ADRPRVEPRAQRAVVTESSLYGEHLA 373

* . * *

NP_001138615.1 --PG----VKEPEVSAGQMPGPGEAELTGGSEWTRAQ-----REHEAQKQHSVELWVQFS 376

AAP35868.1 HMGGPQDFEKSPRDRCSPPPPPREASLAVCGLLQELSSIRQFLEKRDEIREVARDWLRVG 452

EAW67236.1 QPGT------------------------LKEVWSQLQSISNYLQTQDQTDQQEAEWLVLL 409

. . : . : . *: .

NP_001138615.1 HAMDALLFRLYLLFMASSIITVICLWNT---- 404

AAP35868.1 SVLDKLLFHIYLLAVLAYSITLVMLWSIWQYA 484

EAW67236.1 SRFDRLLFQSYLFMLGIYTITLCSLWALWGGV 441

:* ***: **: : **: **

**Supplementary Figure 4** Clustal Omega alignment of human 5-HT_3_ receptor subunits 5HT3A (AAP35868.1), 5HT3B (EAW67236.1), 5HT3D isoform 1 precursor (NP_001138615.1). The highlights represent: Cys-loop (yellow), TM1 (green), TM2 (cyan), TM3 (pink), and TM4 (red) and loops in extra cellular region (grey). The order of the extracellular loops in grey from amino terminal to carboxy terminal is as follows D, A, E, B, F, and C. Conserved F and T residues in TM2 lining are in red. The asterisk (*), colon (:), and dot (.) indicate identical amino acid residues, conserved substitutions, and semi-conserved substitutions across all sequences used in the alignment, respectively.


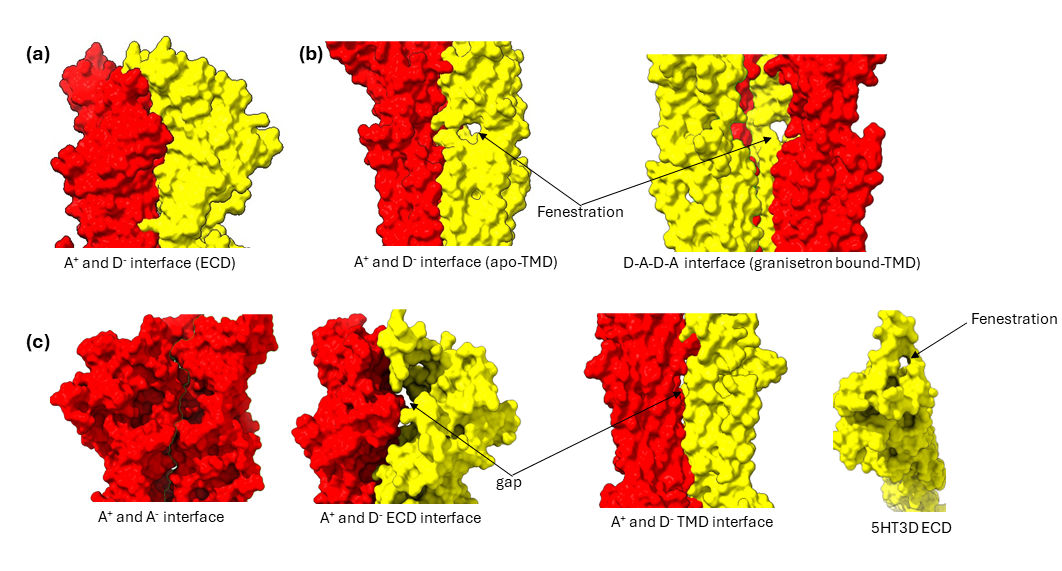


**Supplementary Figure 5** Interface of heteromeric 5-HT_3_AD receptors. 5-HT_3_AD receptor heteromer formed with three A (red) subunits and two D (yellow) subunits with AADAD stoichiometry. **(a)** Interface of subunit A and D apo conformation receptor showing no space between 5-HT_3_AD receptor heteromer in the extracellular domain (ECD). **(b)** Apo conformation and granisetron bound conformation of 5-HT_3_AD receptor heteromer showing fenestrations in subunit D transmembrane domain (TMD). **(c)** Interface of A+ A- and A+ D- serotonin (5-HT) bound conformation of 5-HT_3_AD receptor heteromer showing gap between A+ - D- interface and fenestration in subunit D extracellular domain (ECD) and transmembrane domain (TMD).


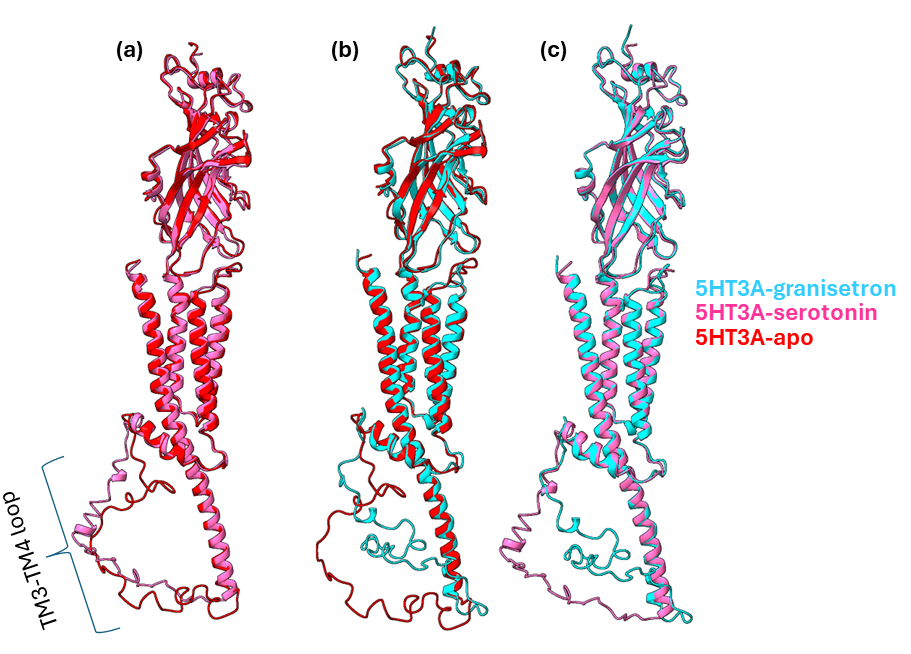


**Supplementary Figure 6** Comparison of conformational changes in subunit A of apo, granisetron and serotonin bound conformations. Overlays of 5HT3A apo (red) and 5-HT (hot pink) **(a)**; 5HT3A granisetron (blue) and apo (red) **(b)**; and granisetron (blue) and serotonin (hot pink) **(c)** bound conformations.


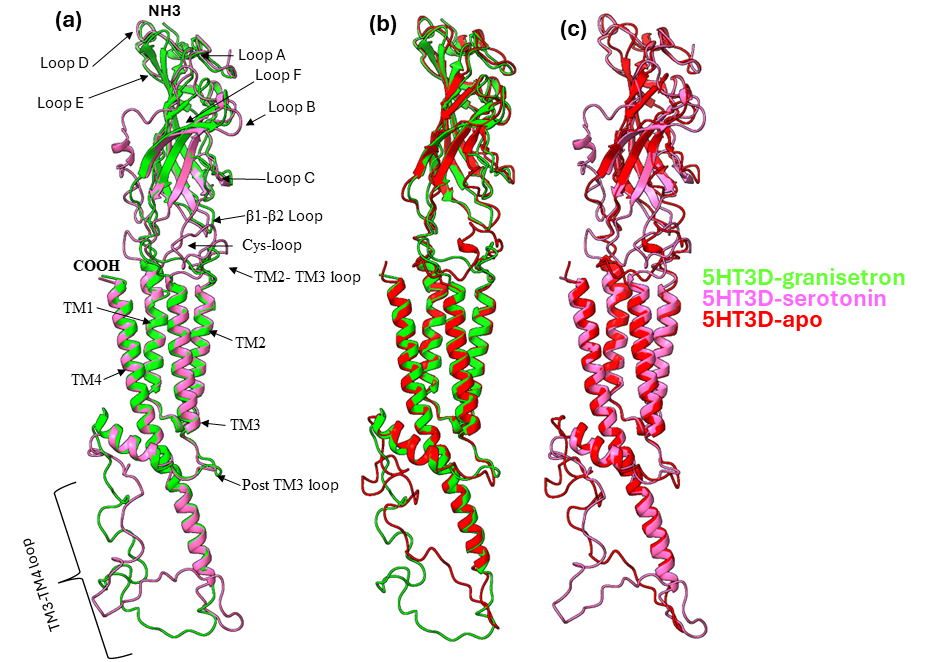


**Supplementary Figure 7** Comparison of conformational changes in homology models of subunit D. **(a)** Overlay of 5HT3D granisetron (green) and 5-HT (magenta) bound conformations with loops and domains noted; **(b)** 5HT3D granisetron (green) bound and ligand free (apo, red) conformations; and **(c)** 5HT3D apo (red) and serotonin (hot pink) bound conformations.


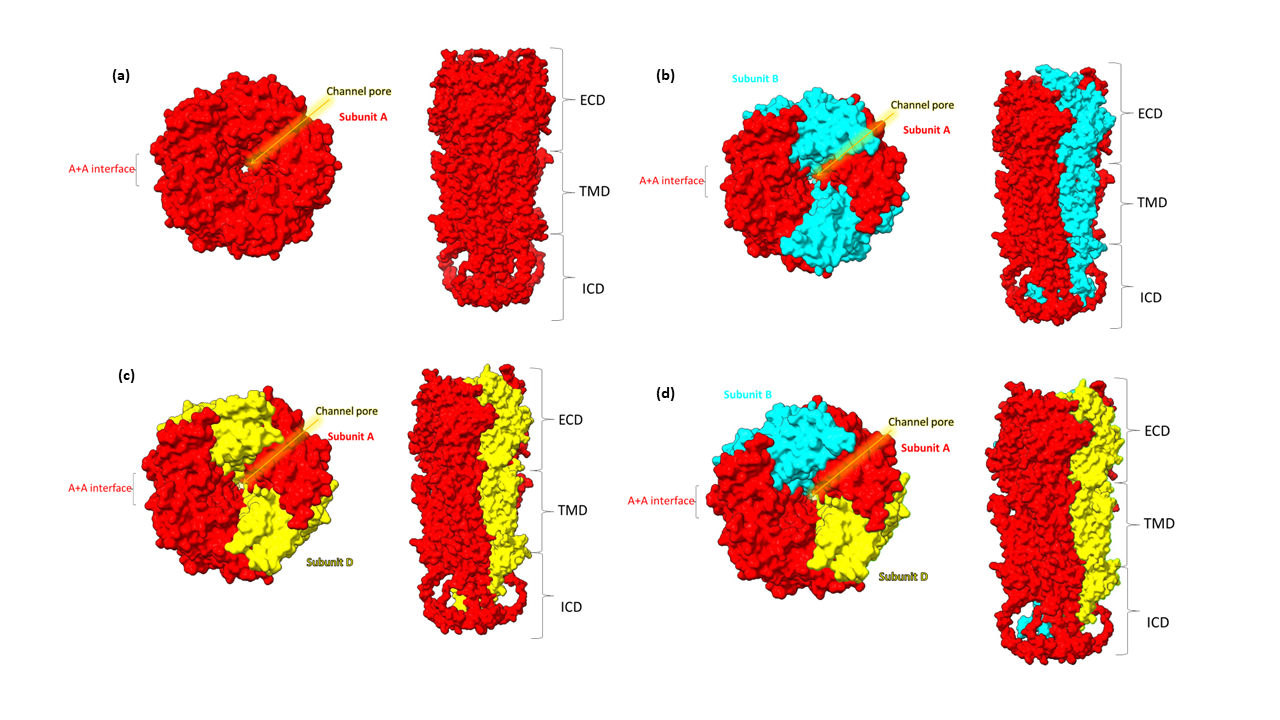


**Supplementary Figure 8** Comparison of heteromeric 5-HT_3_ receptors. **(a)** Perpendicular and transmembrane views showing 5-HT_3_A receptor homomer formed with five A (red) subunits**; (b)** 5-HT_3_AB receptor heteromer formed with three A (red) subunits and two B (cyan) subunits with AABAB stoichiometry**;**  **(c)** 5-HT_3_AD receptor heteromer formed with three A (red) subunits and two D (yellow) subunits with AADAD stoichiometry**;** and **(d)** 5-HT_3_ABD receptor heteromer formed with three A (red) subunits, one B (cyan) and one D (yellow) subunits with AABAD stoichiometry. A+A- interface (ligand binding region) is indicated and extracellular, transmembrane, and intracellular domains denoted as ECD, TMD and ICD respectively.


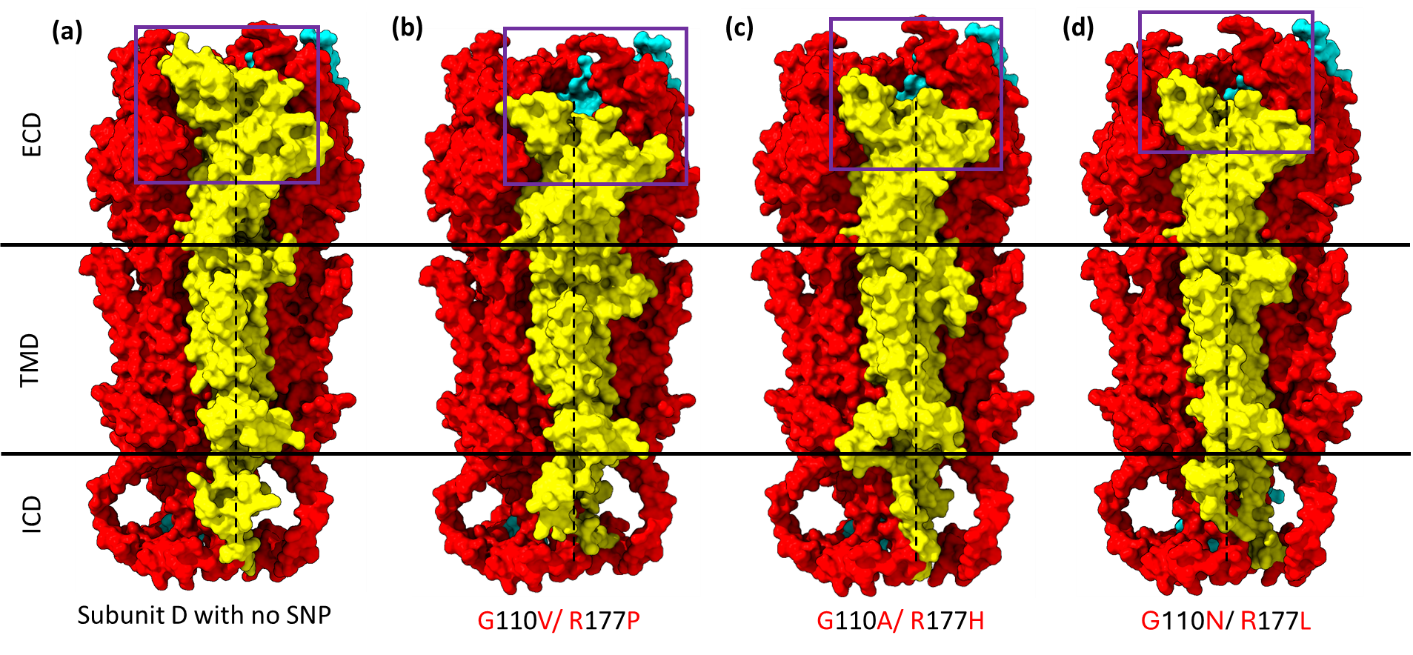


**Supplementary Figure 9** Comparison of ABD heteromer with subunit D SNP substituted models. **(a)** 5-HT_3_ABD heteromer with subunit D with no SNP. **(b)** 5-HT_3_ABD heteromer with SNP G110V / R177P containing subunit D. **(c)** 5-HT_3_ABD heteromer with SNP G110A / R177H containing subunit D. **(d)** 5-HT_3_ABD heteromer with SNP G110N / R177L containing subunit D. Structural changes in extracellular domains of subunit D are highlighted in the purple box and the axis of subunit D and receptors are denoted with black dotted lines.
