# Supplementary material for: Predicted conformations of 5-HT3 receptor ion channels are modified by subunit D
Source: Comput Struct Biotechnol J. 2025 May 29;27:2394–402. doi: 10.1016/j.csbj.2025.05.048 (PMC12172986; doi:10.1016/j.csbj.2025.05.048)
Supplement: Supplementary file 2 — Supplementary material [file mmc2.docx]

**Supplementary Tables for**

**Predicted conformations of 5-HT3 receptor ion channels are modified by subunit D**

**Santosh T. R. B. Rao^1,2^, Helen R. Irving^1,2^**

^1^ La Trobe Institute for Molecular Science, La Trobe University, Bendigo Vic 3550, Australia.

^2^ Holsworth Biomedical Research Centre, Department of Rural Clinical Sciences, La Trobe Rural Health School, La Trobe University, Bendigo Vic 3550, Australia.

**List of Tables**

**Supplementary Table 1** 5HT3D subunit variant details obtained from NCBI database.

**Supplementary Table 2** 5HT3A, B and D subunit details based on structures of the mouse 5HT3A subunits 6np0.1.A  [1]; 6dg7.1.A  and 6be1.1.A  [2].

**Supplementary Table 3** Channel pore dimensions in different ligand bound conformations of models of 5-HT_3_A, 5-HT_3_AB_,_ 5-HT_3_AD_,_ and 5-HT_3_ABD heteromeric receptors.

**Supplementary Table 4** Non-synonymous SNPs and their possible amino acid substitutions in the extracellular loop in HTR3D subunit isoforms.

**References cited**

**Supplementary Table 1** 5HT3D subunit variant details obtained from NCBI database.

| **Gene ID** | **Transcript ID** | **Translation ID** | **Symbol** | **Transcript** | **Length (nt)** | **Protein** | **Length (aa)** | **Isoform** | **UniProt Match** |
| --- | --- | --- | --- | --- | --- | --- | --- | --- | --- |
| 200909 | ENST00000334128.6 | ENSP00000405409.2 | *HTR3D*-201 | NM_182537.3 | 1459 | NP_872343.2 | 279 | 2 | Q70Z44-4 |
| 200909 | ENST00000428798.7 | ENSP00000371929.3 | *HTR3D*-203 | NM_001145143.1 | 1609 | NP_001138615.1 | 404 | 1 precursor | Q70Z44-1 |
| 200909 | ENST00000453435.1 | ENSP00000389268.1 | *HTR3D*-204 | XM_017005854.2 | 1212 | XP_016861343.1 | 233 | X1 | Q70Z44-3 |
| 200909 | ENST00000382489.3 | ENSP00000334315.2 | *HTR3D*-202 | NM_001163646.2 | 1759 | NP_001157118.1 | 454 | 3 precursor | F6WC43 |

aa: amino acid; nt: nucleotides

**Supplementary Table 2** 5HT3A, B and D subunit details based on structures of the mouse 5HT3A subunits 6np0.1.A  [1]; 6dg7.1.A  and 6be1.1.A  [2].

| **Target** | **Template** | **GMQE** | **QMEANDisCo Global** | **identity** |
| --- | --- | --- | --- | --- |
| Human 5HT3A (AAP35868.1) | 6np0.1. A (Cryo-EM structure of 5-HT_3_A receptor in presence of granisetron) | 0.73 | 0.72 ± 0.05 | 87.17% |
|  | 6be1.1. A (5-hydroxytryptamine receptor 3A) | 0.68 | 0.66 ± 0.05 | 87.14% |
|  | 6dg7.1. A (5-HT_3_A receptor in a serotonin-bound conformation- State 1) | 0.73 | 0.68 ± 0.05 | 87.18% |
| Human 5HT3B (EAW67236.1) | 6np0.1. A (Cryo-EM structure of 5-HT_3_A receptor in presence of granisetron) | 0.66 | 0.68 ± 0.05 | 45.78% |
|  | 6be1.1. A (Cryo-EM structure of mouse serotonin receptor) | 0.62 | 0.62 ± 0.05 | 45.76% |
|  | 6dg7.1. A (mouse 5-HT_3_A receptor in a serotonin-bound conformation- State 1) | 0.73 | 0.68 ± 0.05 | 44.70% |
| Human 5HT3D (NP_001157118.1) | 6np0.1. A (Cryo-EM structure of 5-HT_3_A receptor in presence of granisetron) | 0.49 | 0.50 ± 0.05 | 34.38% |
|  | 6be1.1. A (Cryo-EM structure of mouse serotonin receptor) | 0.47 | 0.47 ± 0.05 | 34.20% |
|  | 6dg7.1. A (mouse 5-HT_3_A receptor in a serotonin-bound conformation- State 1) | 0.36 | 0.37 ± 0.05 | 34.03% |

**Supplementary Table 3** Channel pore dimensions in different ligand bound conformations of models of 5-HT_3_A, 5-HT_3_AB_,_ 5-HT_3_AD_,_ and 5-HT_3_ABD heteromeric receptors. The distance (Å) was measured between the amino acids at the interface of opposing A subunits in extracellular (EC) domain (A^+^ (Lys 136) - A^-^ (Lys 136)), transmembrane (TM) domain (A^+^ (Glu 278) - A^-^ (Glu 278)), intracellular domain (IC) (A^+^ (Leu 424) - A^-^ (Leu 424)).

|  |  | **Pore diameter (Å)** | | |
| --- | --- | --- | --- | --- |
| **Receptor stoichiometry** | **ligand bound** | **EC domain** | **TM domain** | **IC domain** |
| 5-HT_3_A-A-A-A-A | Apo (ligand free) | 11.154 | 6.879 | 8.044 |
|  | Granisetron bound | 8.879 | 12.212 | 7.216 |
|  | Serotonin bound | 9.957 | 17.279 | 9.549 |
| 5-HT_3_A-A-B-A-B | Apo (ligand free) | 11.160 | 7.996 | 8.133 |
|  | Granisetron bound | 9.311 | 13.850 | 6.856 |
|  | Serotonin bound | 10.011 | 17.393 | 10.980 |
| 5-HT_3_A-A-D-A-D | Apo (ligand free) | 11.160 | 6.897 | 8.133 |
|  | Granisetron bound | 9.311 | 12.775 | 6.586 |
|  | Serotonin bound | 10.011 | 17.393 | 10.070 |
| 5-HT_3_A-A-B-A-D | Apo (ligand free) | 10.938 | 7.966 | 8.138 |
|  | Granisetron bound | 9.311 | 12.775 | 6.586 |
|  | Serotonin bound | 10.111 | 16.934 | 10.400 |
| Summary of size differences (largest to smallest) | Apo (ligand free) | AB=AD>A>ABD | ABD=AB>AD>A | ABD>AD=AB>A |
|  | Granisetron bound | AB=ABD=AD>A | AB>ABD=AD>A | A>AB>AD=ABD |
|  | Serotonin bound | ABD>AD=AB>A | AB=AD>A>ABD | AB>ABD>AD>A |

**Supplementary Table 4** Non-synonymous SNPs and their possible amino acid substitutions in the extracellular loop in HTR3D subunit isoforms.

| Variant | Amino acid position for SNP_rs1000952 | Amino acid position for SNP_rs6443930 | Reference |
| --- | --- | --- | --- |
| NP_872343.2 | Arg 52 His | Gly 36 Ala | [3-5] |
|  | Arg 52 Pro | Gly 36 Val |  |
|  | Arg 52 Leu | Gly 36 Asp |  |
| NP_001157118 | Arg 225 His |  |  |
|  | Arg 225 Pro |  |  |
|  | Arg 225 Leu |  |  |
| NP_001138615.1 | Arg 177 His | Gly 110 Ala |  |
|  | Arg 177 Pro | Gly 110 Val |  |
|  | Arg 177 Leu | Gly 110 Asp |  |
| NP_016861343 | Arg 6 His |  |  |
|  | Arg 6 Pro |  |  |
|  | Arg 6 Leu |  |  |

**References:**

1. Basak S, Gicheru Y, Kapoor A, Mayer ML, Filizola M, Chakrapani S. Molecular mechanism of setron-mediated inhibition of full-length 5-HT(3A) receptor. Nature Communications 2019;10(1):3225.

2. Basak S, Gicheru Y, Samanta A, Molugu SK, Huang W, Fuente M, et al. Cryo-EM structure of 5-HT(3A) receptor in its resting conformation. Nature Communications 2018;9(1):514.

3. Kim HW, Kang JI, Lee SH, An SK, Sohn SY, Hwang EH, et al. Common variants of HTR3 genes are associated with obsessive-compulsive disorder and its phenotypic expression. Scientific Reports. 2016;6:32564.

4. Singh KP, Dhruva AA, Flowers E, Kober KM, Miaskowski C. A review of the literature on the relationships between genetic polymorphisms and chemotherapy-induced nausea and vomiting. Critical Reviews in Oncology/Hematology. 2018;121:51-61.

5. Deng M, Wang Y, Yu S, Fan Q, Qiu J, Wang Z, et al. Exploring Association Between Serotonin and Neurogenesis Related Genes in Obsessive-Compulsive Disorder in Chinese Han People: Promising Association Between DMRT2, miR-30a-5p, and Early-Onset Patients. Frontiers in Psychiatry. 2022;13:857574.
